# The effect of proportional assist ventilation on the electrical activity of the human diaphragm during exercise

**DOI:** 10.1113/EP090808

**Published:** 2022-11-24

**Authors:** Emily A. M. Gerson, Paolo B. Dominelli, Michael G. Leahy, Shalaya Kipp, Jordan A. Guenette, Bruno Archiza, Andrew William Sheel

**Affiliations:** ^1^ School of Kinesiology The University of British Columbia Vancouver British Columbia Canada; ^2^ Department of Kinesiology University of Waterloo Waterloo Ontario Canada; ^3^ Centre for Heart Lung Innovation Providence Research The University of British Columbia, St. Paul's Hospital Vancouver British Columbia Canada; ^4^ Department of Physical Therapy Faculty of Medicine The University of British Columbia Vancouver British Columbia Canada; ^5^ Department of Physiotherapy Cardiopulmonary Physiotherapy Laboratory Nucleus of Research in Physical Exercise, Federal University of Sao Carlos Sao Carlos Brazil

**Keywords:** oesophageal pressure, respiratory mechanics, ventilatory assist, work of breathing

## Abstract

We hypothesized that when a proportional assist ventilator (PAV) is applied in order to reduce the pressure generated by the diaphragm, there would be a corresponding reduction in electrical activity of the diaphragm. Healthy participants (five male and four female) completed an incremental cycle exercise test to exhaustion in order to calculate workloads for subsequent trials. On the experimental day, participants performed submaximal cycling, and three levels of assisted ventilation were applied (low, medium and high). Ventilatory parameters, pulmonary pressures and EMG of the diaphragm (EMG_di_) were obtained. To compare the PAV conditions with spontaneous breathing intervals, ANOVA procedures were used, and significant effects were evaluated with a Tukey–Kramer test. Significance was set at *P* < 0.05. The work of breathing was not different between the lowest level of unloading and spontaneous breathing (*P* = 0.151) but was significantly lower during medium (25%, *P* = 0.02) and high (36%, *P* < 0.001) levels of PAV. The pressure–time product of the diaphragm (PTP_di_) was lower across PAV unloading conditions (*P* < 0.05). The EMG_di_ was significantly lower in medium and high PAV conditions (*P* = 0.035 and *P* < 0.001, respectively). The mean reductions of EMG_di_ with PAV unloading were 14, 22 and 39%, respectively. The change in EMG_di_ for a given lowering of PTP_di_ with the PAV was significantly correlated (*r* = 0.61, *P* = 0.01). Ventilatory assist during exercise elicits a reduction in the electrical activity of the diaphragm, and there is a proportional lowering of the work of breathing. Our findings have implications for exercise training studies using assisted ventilation to reduce diaphragm work in patients with cardiopulmonary disease.

## Abstract (highlights)

- **What is the central question of this study?**
  What is the effect of lowering the normally occurring work of breathing on the electrical activity and pressure generated by the diaphragm during submaximal exercise in healthy humans?
- **What is the main finding and its importance?**
  Ventilatory assist during exercise elicits a proportional lowering of both the work performed by the diaphragm and diaphragm electrical activity. These findings have implications for exercise training studies using proportional assist ventilation to reduce diaphragm work in patients with cardiopulmonary disease.

## 1 INTRODUCTION

With dynamic exercise, the work of breathing (WOB) increases exponentially as a function of minute ventilation (${\dot{V}}_{E}$), and the O_2_ uptake of the respiratory muscles can represent upwards of ∼10–15% of whole‐body maximal O_2_ uptake (Aaron et al., 1992; Dominelli et al., 2015). Findings from experimental animal studies show that vascular conductance and blood flow to the respiratory muscles increase several‐fold and to an extent similar to that of the locomotor muscles (Manohar, 1986). To understand the relationship between the high demand for blood flow and O_2_ delivery to respiratory and locomotor muscles during exercise, in a series of investigations the work of the inspiratory muscles was lowered experimentally during exercise (Sheel et al., 2020). Briefly, ‘unloading’ the respiratory muscles of healthy young individuals with a proportional assist ventilator prevents diaphragm fatigue, attenuates end‐exercise locomotor muscle fatigue, increases exercise duration and reduces ratings of dyspnoea. With respect to blood flow distribution, reduction of the normally occurring WOB that accompanies heavy exercise results in an increase in limb vascular conductance and blood flow despite a concomitant reduction in stroke volume and cardiac output (Harms et al., 1997, 1998). When a high WOB is isolated to diaphragm work, there is a time‐dependent increase in muscle sympathetic nerve activity in the resting limb (St. Croix et al., 2000) coupled with functional consequences in the form of vasoconstriction and reduced blood flow in the inactive limb (Sheel et al., 2001). These findings provide evidence of a metabolite‐induced reflex originating in the human diaphragm, which is analogous to that emanating from limb locomotor muscles working at high intensity (Mitchell et al., 1983).

There are several considerations when interpreting the above‐mentioned body of work. First, lowering the work performed by the diaphragm during submaximal rather than strenuous or near‐maximal exercise has received less attention (Dominelli et al., 2019; Wetter et al., 1999). Understanding the contractile activity of the diaphragm is relevant during submaximal exercise given that there are instances when a relatively high WOB is sustained (e.g., during exercise in healthy ageing, prolonged endurance‐type exercise or exposure to a hypoxic environment). Furthermore, patients with cardiopulmonary disease, such as heart failure (Olson et al., 2010) or chronic obstructive pulmonary disease (Cherniack, 1959), demonstrate a disproportionately high WOB during submaximal exercise. Second, the typical experimental approach for proportional assist ventilation (PAV)‐related studies has been to maximize the degree of ventilatory assistance that the participant will tolerate such that the WOB is reduced as much as possible. ‘Maximally unloading’, however, results in inter‐individual variability of ‘acceptance’ of the ventilatory assist and thus the experimental variable. In turn, this variability will affect the WOB and contraction of the diaphragm depending on the degree of ventilatory assist provided. Third, it is unclear whether unloading impacts the different respiratory muscles equally across different exercise intensities and levels of unloading. The basis for this question lies, in part, in the differential recruitment of respiratory muscles throughout graded exercise (Aliverti et al., 1997; Goldman et al., 1976; Molgat‐Seon et al., 2018).

Based on the above, we sought to gain a better understanding and to quantify the effects of experimentally lowering the normally occurring WOB on the electrical activity and pressure generated by the diaphragm during submaximal exercise. We hypothesized that when ventilatory assist is used during submaximal exercise, the reductions in pressure generated by the diaphragm would be proportional to reductions in electrical activity of the diaphragm. We hypothesized further that the level of ventilatory assist would result in a dose‐dependent lowering of diaphragm electrical activity.

## 2 METHODS

### 2.1 Ethical approval

This study was approved by the Clinical Research Ethics Board at The University of British Columbia (approval no. H18‐03414) and conforms with Canada's Tri‐Council Statement for the ethical conduct of research involving humans, consistent with the *Declaration of Helsinki*, except for registration in a database. All participants provided written informed consent.

### 2.2 Participants

Nine active, healthy participants (five male and four female) were recruited and were non‐smoking, had no history of asthma or other physician‐diagnosed cardiorespiratory disease and were excluded if they presented any contraindications to performing exercise (physical activity readiness questionnaire for everyone; PAR‐Q+). Participants regularly participated in aerobic physical activity (minimum of three times per week, ≥30 min). Despite familiarization trials, repeated instruction and feedback regarding breathing during unloading, two participants were unable to accept ventilatory assist satisfactorily during the PAV exercise trials, and all their results were removed from analysis (*n* = 7; four male and three female). Descriptive participant characteristics are shown in Table 1.

**TABLE 1:** Descriptive characteristics and maximal exercise data

**Parameter**	**All (*n* = 7)**	**Male (*n* = 4)**	**Female (*n* = 3)**
**Descriptive characteristics**			
Age (years)	26.4 ± 1.0	25.8 ± 3.5	27.3 ± 1.5
Height (cm)	173.7 ± 4.9	175.8 ± 2.7	171.5 ± 6.5
Weight (kg)	68.1 ± 7.0	72.1 ± 6.4	62.8 ± 5.8
BMI (kg m^−2^)	22.5 ± 1.5	23.3 ± 1.4	21.5 ± 0.9
**Maximal exercise**			
Heart rate (beats min^−1^)	182 ± 10	182 ± 5	177 ± 16
${\dot{V}}_{O_{2}}$ (L min^−1^)	3.57 ± 0.63	3.96 ± 0.30	3.16 ± 0.65
${\dot{V}}_{O_{2}}$ (ml kg^−1^ min^−1^)	53.1 ± 8.0	55.8 ± 7.6	50.9 ± 8.5
${\dot{V}}_{CO_{2}}$ (L min^−1^)	4.07 ± 0.71	4.53 ± 0.39	3.59 ± 0.67
RER	1.14 ± 0.06	1.13 ± 0.02	1.14 ± 0.09
${\dot{V}}_{E}$ (L min^−1^)	161 ± 25	163 ± 28	159 ± 25
*V*_T_ (L)	2.77 ± 0.55	3.10 ± 0.43	2.67 ± 0.81
ƒ_b_ (breaths min^−1^)	58 ± 11	55 ± 8	61 ± 15
V˙E/V˙EV˙O2V˙O2	42.9 ± 4.7	40.7 ± 4.7	45.9 ± 3.2
V˙E/V˙EV˙CO2V˙CO2	37.8 ± 5.9	35.7 ± 4.4	40.7 ± 6.3
Work rate (W)	297 ± 21	305 ± 19	286 ± 23
GET (L min^−1^)	2.00 ± 0.39	2.41 ± 0.42	1.63 ± 0.31
10% < GET WL (W)	146 ± 42	175 ± 32	108 ± 31

*Note*: Values are means ± SD.

Abbreviations: BMI, body mass index; ƒ_b_, breathing frequency; GET, gas exchange threshold; RER, respiratory exchange ratio; ${\dot{V}}_{CO_{2}}$, rate of carbon dioxide production; ${\dot{V}}_{E}$, minute ventilation; ${\dot{V}}_{O_{2}}$, rate of O_2_ uptake, *V*_T_, tidal volume; 10% < GET WL, 10% below gas exchange threshold workload.

### 2.3 Experimental overview

Participants completed two testing sessions, which were separated by a minimum of 48 h. On the first day, participant characteristics were determined, followed by an incremental exercise test on a cycle ergometer (Velotron: Racer Mate, Seattle, WA, USA) in order to determine whole‐body maximal O_2_ uptake and calculate workloads for the subsequent experimental day. Males and females began the maximal exercise test at 120 and 80 W, respectively, and work rate increased in a stepwise fashion by 20 W every 2 min for both sexes. The maximal test was terminated when cadence dropped below 60 rpm despite verbal encouragement. Cardiorespiratory variables were assessed continuously during exercise as previously described (Dominelli et al., 2011). After completion of the maximal exercise test, participants were familiarized with PAV during exercise to ensure that they were accustomed to the sensation of assisted breathing. Specifically, participants were coached to relax and allow the proportional assist ventilator to assist inspiration while maintaining a consistent cycling cadence.

On the experimental day, participants completed a continuous cycle test at a work rate corresponding to 10% less than their gas exchange threshold (10% < GET) (Schneider et al., 1993) determined from the first session. To ensure participant‐to‐ventilator synchronicity from the start of the experiment, the protocol began after ∼1 min of cycling, when participants successfully accepted 10 breaths with PAV unloading. Each participant followed the same sequence during the experimental trials (PAV low; spontaneous; PAV medium; spontaneous; PAV high; and spontaneous) and spent ∼1 min in each segment for a total trial length of 6 min. The PAV operator determined the magnitude of unloading for each participant via the presence or absence of signs of discomfort and the ability to accept positive pressure unloading. The PAV unloading condition magnitude (low, medium and high) was participant specific based on the amount of pressure unloading the participant could tolerate. As the protocol progressed to another unloading segment, the operator would gradually increase the level of PAV unloading; as such, the unloading in the PAV low was less than the PAV medium condition, and the PAV high condition was the highest unloading. The PAV was not operational during spontaneous breathing intervals and did not provide ventilatory assistance or added resistance. Ventilatory parameters, oesophageal and gastric pressures along with EMG measures of the diaphragm (EMG_di_), scalenes (EMG_sca_) and sternocleidomastoid (EMG_scm_) were measured continuously during exercise.

### 2.4 Flow, pressure and volume

During the PAV trials, the flow, pressure and volume were collected using hardware and software previously described elsewhere (Dominelli, Archiza et al., 2017). In brief, inspiratory and expiratory flow were measured using calibrated pneumotachometers (model 3813; Hans Rudolph, Kansas City, MO, USA) connected to a two‐way non‐rebreathing valve (model 2700; Hans Rudolph). The expired pneumotachometer was heated to 37°C, whereas inspiratory air was left at ambient temperature. Expired gas was sampled via a port at the end of a mixing chamber (ML 206; ADInstruments, Dunedin, New Zealand). Mouth pressure was measured at a port located in the mouthpiece (DP15‐34; Validyne Engineering, Northridge, CA, USA), and the oesophageal and gastric pressures were obtained with a balloon catheter system (Guangzhou Yinghui Medical Equipment, Guangzhou, China) (see section 2.5 Electromyography) connected to pressure transducers (model DP15‐34; Validyne Engineering), which were calibrated before and after each test using a digital manometer (2021P; Digitron, Torquay UK). Before balloon catheter placement, a topical anaesthetic (lignocaine hydrochloride) was used to minimize participant discomfort. Placement of the oesophageal and gastric balloon catheters was performed as we have previously described (Molgat‐Seon et al., 2018), and they were filled with 1 and 2 ml of air, respectively. Flow, pressure, tidal volume (*V*_t_), ${\dot{V}}_{E}$, oesophageal (*P*_oes_) and gastric (*P*_ga_) pressures were recorded continuously at 2 kHz, with an analog‐to‐digital converter (ADInstruments, Colorado Springs, CO, USA) running associated software (LabChart Pro v.8.1), and transdiaphragm pressure was (*P*_di_) was calculated.

### 2.5 Electromyography

Electromyography of the scalenes (SCA) and sternocleidomastoid (SCM) was assessed using surface electrodes (3M Red Dot 2256‐50; 3M, Saint Paul, MN, USA). These two muscles were studied because they are they are the two most prominent inspiratory muscles in the neck, they contribute significantly to inspiratory pressure (Legrand et al., 2003), and they are accessible for the purposes of EMG recordings in humans. Placement of the electrodes for the SCM was on the long axis between the medial clavicle and mastoid process. Placement for the SCA electrode was at the level of the cricoid cartilage in the posterior triangle of the participant's neck (Chiti et al., 2008).

Placement of the EMG_di_ catheter was optimized as described elsewhere (Luo et al., 2008). Raw EMG_di_ signals were amplified and processed through a notch filter at 60 Hz (RA‐8; Yinghui Medical Technology, Guangzhou, China). All EMG signals were processed further through a bandpass filter between 10 Hz and 3 kHz, and filtered data were transformed to a root mean square using a 0.1 s moving average window.

All EMG_di_, EMG_scm_ and EMG_sca_ used in analysis were selected during periods of inspiration and were free of cardiac artefact based on visual inspection. Give that the EMG_di_ was measured using a multi‐paired catheter (to account for changes during lung volumes during exercise and posture), the highest electrode pair that yielded the greatest EMG activity for a specific breath was used in the analysis. Data from the highest pair were then expressed as a percentage relative to the participant's maximal activation from an inspiratory capacity (IC) manoeuvre, sniff or maximal inspiratory pressure (MIP) manoeuvre performed at rest (Molgat‐Seon et al., 2018). Participants performed IC manoeuvres by inhaling maximally after a full exhalation. Sniffs were a series of short, maximal sniffs. Maximal inspiratory pressure manoeuvres were performed from residual volume, followed by a maximal inspiratory effort against a semi‐occluded mouthpiece. The EMG_scm_ and EMG_sca_ data were also expressed as percentages relative to the participant's highest IC, sniff or MIP.

### 2.6 Respiratory mechanics

Respiratory muscle work was quantified by two methods to account for different aspects of mechanical work. To assess total respiratory system work or WOB, we integrated composite average oesophageal pressure–volume loops from ∼8 breaths, during which physiological artefacts (i.e., cough and swallow) were absent (Dominelli et al., 2016). To quantify the work performed by the diaphragm and total system separately, we calculated the transdiaphragmatic pressure–time product (PTP_di_) and oesophageal pressure–time product (PTP_oes_). The ratio of PTP_di_ to PTP_oes_ was calculated to determine the contribution of the diaphragm to the total inspiratory muscle pressure production for each of the conditions, and the gastric pressure–time product (PTP_ga_) was also calculated.

### 2.7 Statistical analysis

Repeated‐measures ANOVA procedures were used to investigate within‐subject unloading by comparing the PAV unloading conditions (low, medium and high) with spontaneous breathing intervals for all variables. Comparisons were made for lung mechanics and EMG variables. When ANOVA revealed significant effects, a post‐hoc Tukey–Kramer test was performed to identify differences. The level of significance was set at *P* ≤ 0.05 for all tests. Statistical analysis was performed using v.0.12.1 of JASP (Version 0.16.2. JASP Team, 2022, Amsterdam, The Netherlands). A linear regression, accounting for repeated measures within a subject (Bland & Altman, 1995), was performed to determine the association between change in EMG_di_ and change in PTP_di_.

## 3 RESULTS

**FIGURE 1:**
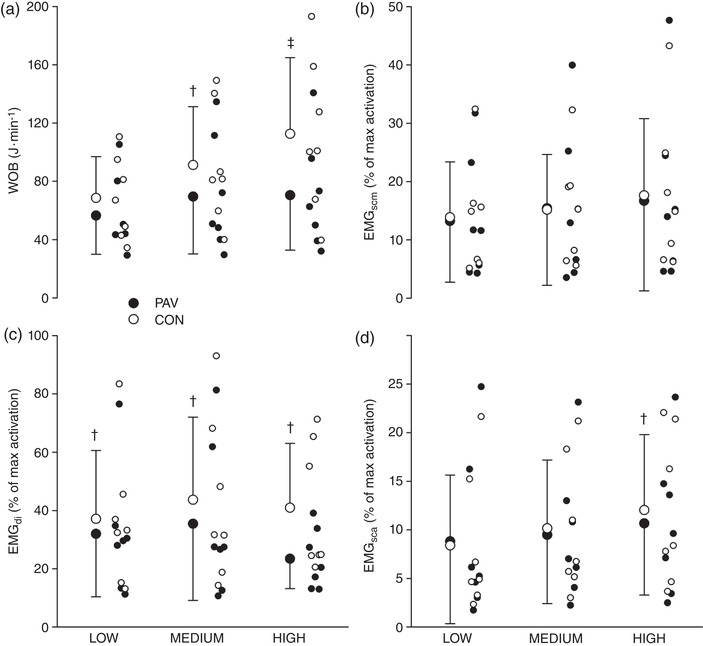
Comparisons of PAV unloading with spontaneous breathing: (a) WOB; (b) EMG_scm_; (c) EMG_di_; and (d) EMG_sca_. Significance was determined through a repeated‐measures ANOVA and Tukey–Kramer test (*n* = 7). Individual data and means ± SD are shown. ^†^ *P* < 0.05 between PAV and spontaneous breathing; ^‡^ *P* < 0.01 between PAV and spontaneous breathing. Abbreviations: CON, control; EMG_di_, EMG of the diaphragm; EMG_sca_, EMG of the scalene; EMG_scm_, EMG of the sternocleidomastoid; PAV, proportional assist ventilation; WOB, work of breathing

**FIGURE 2:**
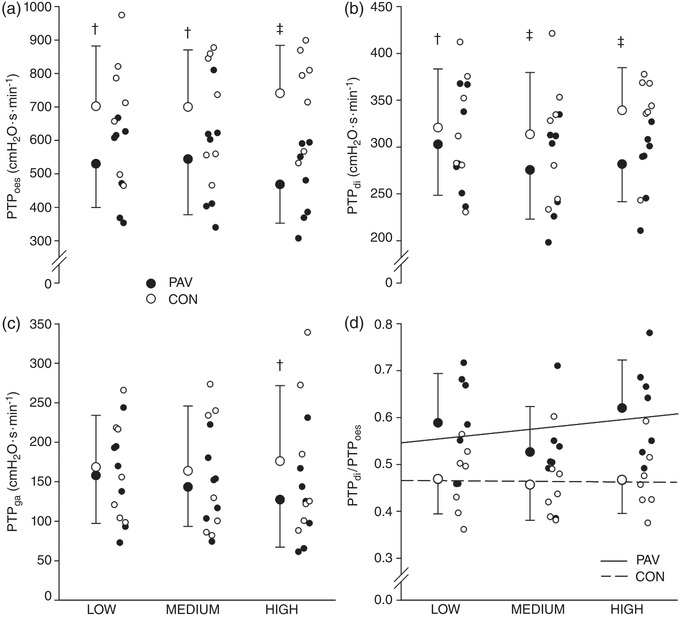
Comparisons of PAV unloading with spontaneous breathing: (a) PTP_oes_; (b) PTP_di_; (c) PTP_ga_; and (d) PTP_di_/PTP_oes_. Significance was determined through a repeated‐measures ANOVA and Tukey–Kramer test (*n* = 7). Individual data and means ± SD are shown. ^†^ *P* < 0.05 between PAV and spontaneous breathing; ^‡^ *P* < 0.01 between PAV and spontaneous breathing. Abbreviations: CON, control; PAV, proportional assist ventilation; PTP_di_, pressure–time product of the diaphragm; PTP_gas_, gastric pressure–time product; PTP_oes_, pressure–time product of oesophagus

Table 2 summarizes group mean values for respiratory mechanics during the experimental protocol, and individual values are depicted in Figures 1 and 2. The WOB was not different between the lowest unloading of the PAV and spontaneous breathing (*P* = 0.151) but was significantly lower during medium and high PAV unloading compared with spontaneous breathing (∼25% mean reduction, *P* = 0.02 and ∼36% mean reduction, *P* < 0.001, respectively). Across all PAV unloading trials compared with spontaneous breathing conditions, there were no statistical differences for ventilation or heart rate (both *P* > 0.05). The PTP_oes_ was significantly lower in all PAV unloading conditions compared with spontaneous breathing (*P* < 0.01). The PTP_di_ was significantly lower in all PAV unloading conditions compared with spontaneous breathing (*P* < 0.05 low comparison; *P* < 0.01 medium and high comparisons). The PTP_ga_ was significantly different only at high PAV unloading compared with spontaneous breathing (*P* < 0.01). No statistical differences were found between any PAV unloading intensities compared with spontaneous breathing for the quotient of PTP_di_ to PTP_oes_ (*P* > 0.05).

**TABLE 2:** Cardiorespiratory variables, work of breathing, diaphragm activation and accessory respiratory muscle activation during experimental protocol

**Parameter**	**PAV (low)**	**Off**	PAV (medium)	**Off**	**PAV (high)**	**Off**
WOB (J min^−1^)	56.6 ± 25.6	68.6 ± 27.0	69.9 ± 38.9	91.2 ± 40.0^a^	70.2 ± 38.1	112 ± 52.4^b^
∆WOB (%)	16.9 ± 14.6		24.6 ± 15.5		35.5 ± 17.7	
${\dot{V}}_{E}$ (L min^−1^)	53.9 ± 6.4	51.4 ± 7	58.5 ± 8.6	57.0 ± 7.9	65.9 ± 8.7	60.3 ± 10.1
*V*_T_ (L)	1.86 ± 0.81	1.87 ± 0.83	2.04 ± 0.83	2.08 ± 0.79	2.16 ± 0.84	1.97 ± 0.76
ƒ_b_ (breaths min^−1^)	29.0 ± 1.9	29.0 ± 2.7	29.7 ± 2	27.3 ± 1.9	30.2 ± 1.5	30.0 ± 2.3
EMG_di_ (%)	32.0 ± 21.5	37.1 ± 23.5	35.4 ± 26.2	43.6 ± 28.4^a^	23.4 ± 10.2	40.9 ± 27.5^b^
∆EMG_di_ (%)	14.0 ± 9.8		21.6 ± 13.3		38.8 ± 15.3	
EMG_scm_ (%)	12.4 ± 10.0	12.7 ± 9.4	14.2 ± 12.7	13.8 ± 9.6	15.2 ± 15	17.5 ± 12.1
EMG_sca_ (%)	9.8 ± 8.3	9.0 ± 6.9	10.0 ± 6.8	10.5 ± 8.4	10.8 ± 5.3	13.6 ± 4.3^a^
PTP_oes_ (cmH_2_O s min^−1^)	530 ± 130.6	702 ± 180.5^a^	544 ± 166.0	700 ± 170.5^a^	468 ± 115.8	741 ± 143.5^b^
∆PTP_oes_ (%)	20.0 ± 14.2		22.6 ± 11.3		36.9 ± 8.6	
PTP_ga_ (cmH_2_O s min^−1^)	158 ± 60.5	169 ± 65.5	143 ± 49.8	164 ± 82.3	127 ± 60.1	176 ± 95.6^b^
PTP_di_ (cmH_2_O s min^−1^)	303 ± 52.0	320 ± 62.7^a^	276 ± 52.6	314 ± 66.1^b^	282 ± 40.0	339 ± 45.6^b^
∆PTP_di_ (%)	8.9 ± 8.8		14.4 ± 11.2		18.1 ± 7.5	
PTP_di_/PTP_oes_	0.58 ± 0.10	0.46 ± 0.07	0.52 ± 0.09	0.45 ± 0.07	0.62 ± 0.10	0.47 ± 0.07

*Note*: Values are means ± SD.

Abbreviations: EMG_di_, EMG of the diaphragm; EMG_sca_, EMG of the scalene; EMG_scm_, EMG of the sternocleidomastoid; ƒ_b_, breathing frequency; PAV, proportional assist ventilation; PTP_di_, pressure–time product of the diaphragm; PTP_gas_, pressure–time product of the gastric pressure; PTP_oes_, pressure–time product of oesophagus; ${\dot{V}}_{E}$, minute ventilation, *V*_T_, tidal volume; WOB, work of breathing; ∆, change.

^a^ *P* < 0.05 between PAV and spontaneous breathing (off).

^b^ *P* < 0.01 between PAV and spontaneous breathing (off).

**FIGURE 3:**
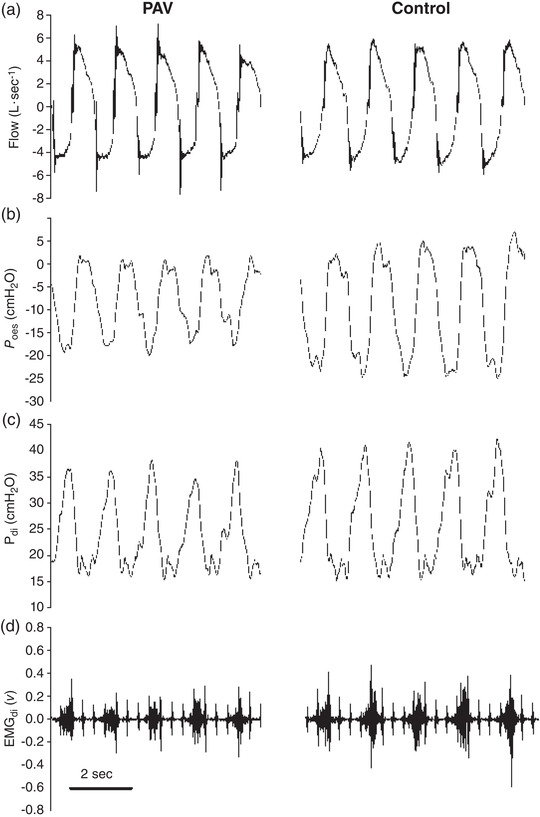
Representative data from a single male participant during the medium unloading trial and corresponding control conditions: (a) flow; (b) PTP_oes_; (c) *P*_di_; and (d) EMG_di_. Shown are five breaths for each section. The average minute ventilation was similar between the PAV (108 L min^−1^) and control (110 L min^−1^) conditions. Note the simultaneous reduction in EMG_di_, *P*_oes_ and *P*_di_ in the PAV conditions, while flow and breathing frequency remained unaltered. Abbreviations: EMG_di_, electrical activity of the fifth transdiaphragmatic electrode pairing on the dual balloon catheter; PAV, proportional assist ventilation; *P*_di_, transdiaphragmatic pressure; *P*_oes_, oesophageal pressure

Figure 3 shows an example of EMG_di_ (fifth pair) and selected respiratory mechanics variables during medium unloading and control conditions; note the PAV‐induced lowering of *P*_oes_ and *P*_di_ and the accompanying reduction in EMG_di_. Group mean values for EMG_di_, EMG_sca_ and EMG_scm_ findings are summarized in Table 2, and individual values are shown in Figure 1. The EMG_di_ was significantly lower in medium and high PAV unloading versus spontaneous breathing conditions (*P* = 0.035 and *P* < 0.001, respectively). Mean reductions of EMG_di_ between PAV unloading and spontaneous breathing were 14, 22 and 39% for low, medium and high PAV, respectively. The EMG_sca_ was not significantly different for both low and medium PAV unloading compared with spontaneous breathing (*P* = 0.5 and *P* = 0.65, respectively). However, EMG_sca_ was significant when comparing high PAV unloading with spontaneous breathing (*P* = 0.02). The EMG_scm_ was unchanged for all three PAV unloading conditions compared with spontaneous breathing (*P* = 0.87, *P* = 0.83 and *P* = 0.17, respectively). When comparing the PAV unloading levels, no significant effect for EMG_sca_ and EMG_scm_ activity was observed (*P* > 0.05).

**FIGURE 4:**
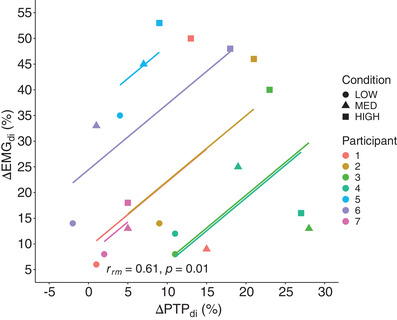
Change in PTP_di_ versus EMG_di_. A linear regression for each participant comparing the change in PTP_di_ with EMG_di_ was determined by using low, medium and high unloading to spontaneous breathing comparisons for PTP_di_ and EMG_di_. Group average *r* = 0.61, *P* = 0.01. Abbreviations: EMG_di_, EMG of the diaphragm; PTP_di_, pressure–time product of the diaphragm; ∆, change

Shown in Figure 4 is the change in EMG_di_ (∆EMG_di_) for a given lowering of PTP_di_ (∆PTP_di_) with PAV across all three trials for individual subjects and the group (*r* = 0.61, *P* = 0.01).

## 4 DISCUSSION

### 4.1 Main findings

The primary purpose of this study was to assess the relationship between the electrical activity and pressure generated by the diaphragm during exercise. To this end, healthy humans performed dynamic submaximal exercise under three levels of ventilatory assist to lower the normally occurring WOB while we measured respiratory mechanics and quantified EMG of the diaphragm and accessory respiratory muscles. Our primary findings are twofold. First, we found that ventilatory assist during submaximal exercise elicits a reduction in the electrical activity of the diaphragm. Second, the level of ventilatory assist resulted in a proportional lowering of both diaphragm electrical activity and the mechanical WOB. Our findings show that parallel increases in the electrical activity and pressure generation of the diaphragm coincide with the hyperpnoea of exercise in humans and that both can be lowered effectively with ventilatory assist in a dose‐dependent manner. Given that cardiopulmonary exercise rehabilitation programmes are often performed at submaximal intensities, our findings have implications for exercise training studies using proportional assist ventilation to reduce diaphragm work in patients with cardiopulmonary disease.

### 4.2 Electrical activity of the respiratory musculature during exercise

In adult humans, there are >60 muscles known to play a role in breathing (i.e., any muscle that assists in lung inflation or deflation; Pilarski et al., 2019). From a functional point of view and with respect to exercise, the respiratory musculature can be categorized more simply into one of three groups: the diaphragm, the rib‐cage muscles and the abdominal muscles (Aliverti et al., 1997). In the present study, we measured the EMG activity of the diaphragm and two rib‐cage muscles (SCA and SCM) simultaneously during submaximal exercise while applying three degrees of ventilatory assist (low, medium and high). In the control or spontaneously breathing conditions, we observed significant EMG_di_ activity (Figure 1c; Table 2), with modest activity of the rib‐cage muscles. Our findings are consistent with the concept that during submaximal exercise the primary muscle of inspiration is the diaphragm, whereas the accessory muscles contribute to lung inflation to a lesser degree (Aliverti et al., 1997; Grassino & Goldman, 1986). As described by Aliverti et al. (1997), with exercise, the diaphragm acts principally as a ‘flow generator’, while the rib cage (and abdominal) muscles develop pressures to move the rib cage and abdomen and serve as ‘pressure generators’. Here, we emphasize that when exercise is performed at higher intensity, rib‐cage muscle activity increases in proportion to ventilatory output (Aliverti et al., 1997; Bye et al., 1984). For example, we have previously shown that lowering the WOB in conditions of heavy exercise (90% of maximal work) reduces electrical activity and muscle perfusion of the SCM (Dominelli, Archiza et al., 2017). Our findings in humans highlight that the accessory muscles of breathing are an important contributor to overall respiratory muscle energetics during heavy exercise and are in agreement with findings in maximally exercising ponies (Manohar, 1990) and rats (Poole et al., 2000).

With increasing levels of ventilatory assist, we observed progressive reductions in EMG_di_ (14, 22 and 39% compared with spontaneous breathing), whereas the degree of PAV unloading had no significant effect on EMG_sca_ and EMG_scm_. We interpret these observations to mean, at least within the context of our experimental conditions, that when humans perform submaximal exercise, the electrical activity of the diaphragm can be lowered effectively with PAV. In contrast, we found no statistical differences in terms of SCA and SCM activity during unloaded breathing. Given that the contribution from the rib‐cage muscles to pressure generation at our modest exercise intensities is relatively low, it is not surprising that our application of PAV did not reduce EMG_sca_ and EMG_scm_. We observed between‐subject variation with respect to EMG_sca_ and EMG_scm_ activity during spontaneous breathing, and application of PAV reduced the activity of these muscles, but on average the reduction was inconsistent or not statistically significant. We show that usage of PAV appears to reduce diaphragm electrical activity effectively, based upon the degree of assistance provided. This dose‐dependent relationship is important when considering that there is between‐subject variation in the degree of assistance a subject is willing to ‘tolerate’ (Dominelli, Molgat‐Seon et al., 2017; Harms et al., 1997; Wetter et al., 1999). Should an experimental goal be to unload the diaphragm maximally (see section 4.4 Perspectives), our findings indicate the need to familiarize participants sufficiently such that the greatest reduction can be applied.

### 4.3 Relationship between EMG_di_ and PTP_di_

We measured diaphragm electrical activation as an estimate of respiratory motor output together with transdiaphragmatic pressure to reflect pressure produced by the diaphragm during contraction (Laveneziana et al., 2019). Using a linear regression that accounted for repeated measures within a subject, we found a close association between PAV‐induced reductions in EMG_di_ and PTP_di_ during exercise (Figure 4). Our approach builds upon previous work and extends our understanding of respiratory muscle contraction during exercise. For example, in healthy resting humans, pressure support reduces transdiaphragmatic pressure and EMG_di_ equally (Sinderby et al., 2007), which agrees with our findings in conditions of high ventilation that accompany dynamic exercise. There is some evidence of an uncoupling between the electrical activity and pressure generation of the diaphragm during incremental exercise in patients with chronic obstructive pulmonary disease (Sinderby et al., 2001). The dissociation between the two variables was associated with dynamic hyperinflation, which reduced diaphragm pressure‐generating capacity and promoted high levels of diaphragm activation. In our study, participants were healthy young adults performing submaximal exercise and unlikely to have developed dynamic hyperinflation, which is in agreement with the association between EMG_di_ and PTP_di_ we observed. In some aerobically trained athletic populations, there can be an increase in end‐expiratory lung volume towards resting values, secondary to expiratory flow limitation (Guenette et al., 2007). Decreases in end‐expiratory lung volume are generally thought to occur in order to optimize diaphragm length and, in turn, minimize the inspiratory work of breathing. It is unknown whether increases in end‐expiratory lung volume towards resting levels in maximally exercising athletic populations result in a weakening or uncoupling of the relationship between EMG_di_ and PTP_di_, and further study is required.

### 4.4 Perspectives

The EMG observations we made, coupled with transdiaphragmatic pressure measurements, provide further insight into the physiological basis for the respiratory muscle metaboreflex (Dempsey et al., 2006). A series of PAV investigations show that by significantly reducing the WOB in healthy subjects during heavy exercise, diaphragm fatigue is prevented, locomotor muscle fatigue at end‐exercise is reduced below control values, exercise duration to exhaustion is extended, and exercising limb vascular conductance and blood flow are increased (Sheel et al., 2018). Reducing respiratory muscle work also results in a lowering of noradrenaline spillover across the exercising leg muscle (Harms et al., 1997) and muscle sympathetic nerve activity of the resting upper limb (Dominelli et al., 2019), suggesting sympathetically mediated vasomotor effects. Most work suggests that a metaboreflex originating in the inspiratory (and/or expiratory) muscles is a major source of this sympathoexcitation. There is a growing body of evidence to show that lowering the WOB in cardiopulmonary patients, such as those with heart failure or chronic obstructive pulmonary disease, has important functional consequences. For example, several different methods to ‘unload’ the respiratory muscles (e.g., PAV, continuous positive airway pressure, inspiratory pressure support, helium–oxygen mixtures) have been shown to increase exercise tolerance (Bianchi et al., 1998; Eves et al., 2006) and perfusion to exercising limbs (Olson et al., 2010; Smith et al., 2020), attenuate locomotor muscle fatigue (Amann et al., 2010) and reduce sensations of breathlessness (O'Donnell et al., 1988; Vogiatzis et al., 2019). The provision of ventilatory assistance during exercise has consistent and measurable effects in both health and disease, highlighting the role that the act of breathing and the respiratory musculature itself have on the integrative nature of large muscle mass exercise. The findings of our study provide additional support to the idea that subtracting respiratory (diaphragm) muscle work elicits reflex effects that originate, at least in part, in the diaphragm. Unlike the results in healthy young individuals, the effects of respiratory muscle unloading are present during relatively mild and submaximal exercise intensities in the above‐mentioned clinical populations. The findings from the present study demonstrate that application of PAV during submaximal exercise effectively lowers the work and electrical activity of the diaphragm. Cardiopulmonary exercise rehabilitation programmes are typically performed at submaximal intensities (Spruit et al., 2013), and our findings show that PAV can effectively lower the work and electrical activity of the diaphragm at these intensities. As such, our results are relevant to rehabilitation investigations where assisted ventilation is used to reduce diaphragm work in conjunction with standard exercise rehabilitation training regimens.

### 4.5 Limitations

Our study was conducted using cycle ergometry, and our interpretation of EMG_di_ and PAV‐related effects are limited to this modality; our results might not necessarily apply to other types of exercise that involve arm movement (i.e., walking, running). Specifically, the diaphragm has separate demands in the upright human for both respiration and stabilization of the lumbar spine (Loring & Mead, 1982), and the non‐ventilatory functions of the diaphragm and other respiratory muscles would need to be considered in conditions of bipedal locomotion with ventilatory assist.

Although the level of ventilatory assist we used was graded, we did not apply a full range of potential unloading (i.e., 0–100%). Potentially, there is a ceiling effect of PAV in terms of diaphragm activity, whereby increasing the amount of ‘assist’ no longer lowers diaphragmatic activity or force output owing to the proportionality and that breathing is still spontaneous; that is, there might be a minimum amount of diaphragm activity required to initiate respiration, without which the proportional assist ventilator cannot operate. The diaphragm also acts to stabilize and resist movements of the abdominal cavity during active expiration (Aliverti et al., 1997). In the present experimental configuration, we did not alter expiratory work, and thus we would predict that this amount of diaphragm activity would be unaltered.

Lastly, it should be emphasized that our study population was young and healthy. Our findings might not necessarily apply to conditions of altered pulmonary mechanics, such as ageing or cardiopulmonary disease, in which respiratory muscle recruitment might differ.

### 4.6 Conclusions

We found that during submaximal exercise the application of ventilatory assist lowered the electrical activity of the diaphragm and that the degree of electrical activity could be modulated by altering the level of ventilatory assist. Our observations of a parallel reduction in the electrical activity and pressure generation of the diaphragm provide good evidence that ventilatory assist is an effective method with which to reduce the demands placed on the diaphragm during exercise. From a clinical perspective, our findings suggest that using PAV is an effective means to reduce diaphragm work and could be considered for future use in patients with cardiopulmonary disease performing exercise. Assisted ventilation during pulmonary rehabilitation might permit patients to perform exercise training at a higher intensity.

## AUTHOR CONTRIBUTIONS

All authors contributed to the conception and design of the work. Emily A. M. Gerson, Paolo B. Dominelli, Michael G. Leahy, Shalaya Kipp, Bruno Archiza and A. William Sheel participated in acquisition and analysis of the work. All authors contributed to the interpretation of the work. All authors drafted the work and revised it critically for important intellectual content. All authors approved the final version of the manuscript and agree to be accountable for all aspects of the work in ensuring that questions related to the accuracy or integrity of any part of the work are appropriately investigated and resolved. All persons designated as authors qualify for authorship, and all those who qualify for authorship are listed.

## CONFLICT OF INTEREST

None declared.

## Supporting information

Statistical Summary Document

## Data Availability

The data supporting the conclusions of this article will be made available by the authors upon reasonable request.
